# Clinical validation of the parent‐report Toronto Obsessive–Compulsive Scale (TOCS): A pediatric open‐source rating scale

**DOI:** 10.1002/jcv2.12056

**Published:** 2021-12-03

**Authors:** Laura J. Lambe, Christie L. Burton, Evdokia Anagnostou, Elizabeth Kelley, Robert Nicolson, Stelios Georgiades, Noam Soreni, Russell J. Schachar, Gregory L. Hanna, Paul D. Arnold, Jennifer Crosbie

**Affiliations:** ^1^ Program of Neurosciences and Mental Health Hospital for Sick Children Toronto Ontario Canada; ^2^ Bloorview Research Institute Holland Bloorview Kids Rehabilitation Hospital Toronto Ontario Canada; ^3^ Department of Pediatrics University of Toronto Toronto Ontario Canada; ^4^ Department of Psychology Queen's University Kingston Ontario Canada; ^5^ Schulich School of Medicine and Dentistry University of Western Ontario London Ontario Canada; ^6^ Department of Psychiatry & Behavioural Neurosciences McMaster University Hamilton Ontario Canada; ^7^ Department of Psychiatry St. Joe's Healthcare Hamilton Ontario Canada; ^8^ Department of Psychiatry University of Toronto Toronto Ontario Canada; ^9^ Department of Psychiatry University of Michigan Ann Arbor Michigan USA; ^10^ The Mathison Centre for Mental Health Research & Education Calgary Alberta Canada; ^11^ Departments of Psychiatry and Medical Genetics, Cumming School of Medicine University of Calgary Calgary Alberta Canada; ^12^ Program of Genetics and Genome Biology Hospital for Sick Children Toronto Ontario Canada

**Keywords:** cross‐disorder phenotyping, neurodevelopmental disorders, obsessive–compulsive disorder, obsessive–compulsive traits, ROC analysis, scale development

## Abstract

**Background:**

There is a need to develop a multipurpose obsessive–compulsive disorder (OCD) measure that is useful for cross disorder research and as a reliable clinical rating scale. The current study examined the psychometric properties and established clinical cutoffs for the parent‐report version of the Toronto Obsessive–Compulsive Scale (TOCS), a 21‐item rating scale of obsessive–compulsive traits.

**Method:**

Participants ranged in age from 6 to 21 years old and had a primary diagnosis of OCD (*n* = 350, 50% female), attention‐deficit/hyperactivity disorder (ADHD) (*n* = 820, 25% female), autism spectrum disorder (ASD) (*n* = 794, 22% female), or were typically developing controls (*n* = 391, 51% female). Confirmatory factor analyses, internal consistency reliability, and convergent and divergent validity of the TOCS were examined in the OCD group. Using various scoring approaches, receiver operating characteristic (ROC) analyses were used to establish a clinical cut‐off by splitting the OCD group into a discovery sample (166 OCD cases, 164 controls) and a validation sample (184 OCD cases, 227 controls). Classification accuracy and TOCS scores were compared across OCD, ADHD, and ASD groups.

**Results:**

The psychometric properties of the TOCS were confirmed. ROC analyses across TOCS scoring approaches in the discovery sample indicated excellent diagnostic discrimination (AUC ≥0.95, sensitivity 77%–92%, specificity 92%–98%). Established cutoffs, when applied in the independent validation sample of OCD cases and controls, showed an overall classification accuracy of 85%–90%. The TOCS total score and symptom count showed good discrimination of OCD from ADHD (AUC ≥0.86) and ASD (AUC ≥0.81). The OCD group scored significantly higher on all TOCS dimensions (except Hoarding) than the ADHD and ASD groups.

**Conclusion:**

The TOCS is a reliable and valid rating scale with strong sensitivity and specificity in discriminating OCD cases from controls, as well as from ASD and ADHD. It is a quantitative OCD measure with important clinical and research applications, with particular relevance for cross disorder phenotyping and population‐based studies.

**Key points**

- The Toronto Obsessive–Compulsive Scale (TOCS) is an open‐source, quantitative measure of pediatric obsessive–compulsive (OC) traits that has been previously validated in pediatric community samples and used to identify the first genome‐wide variant for OC traits
- The current study clinically validated the TOCS, as it has excellent diagnostic discrimination of pediatric obsessive–compulsive disorder (OCD) cases from controls, as well as a strong ability to discriminate OCD cases from attention‐deficit/hyperactivity disorder and autism spectrum disorder cases
- By using the TOCS to measure OC traits, we can gather data to inform both genetic research and clinical practice, thereby minimizing patient burden

## INTRODUCTION

Obsessive–compulsive disorder (OCD) is a psychiatric disorder characterized by recurrent intrusive thoughts or impulses that cause marked distress (obsessions), and repetitive behaviors or mental acts (compulsions). Approximately 50% of individuals with OCD initially developed their symptoms during childhood (Geller, 2006), with pediatric OCD having a particularly strong genetic component (45%–65%; van Grootheest et al., 2005). Like other mental illnesses, OCD may reflect of the extremes of quantitative traits that are normally distributed within the population (Abramowitz et al., 2014; Plomin et al., 2009). This trait‐based conceptualization allows researchers to harness the power of large populations to study the genetic underpinnings of OCD by focusing on quantitative obsessive–compulsive (OC) traits.

The Toronto Obsessive–Compulsive Scale (TOCS) (Burton et al., 2018; Park et al., 2016) is a multidimensional measure that has been designed to measure OC traits in the general population, in addition to being an easily administered rating scale for clinicians. Rating scales are commonly used in clinical settings as a way to identify individuals at the highest need to triage them toward the most appropriate services (e.g., those in need of comprehensive assessment and treatment). Existing rating scales for pediatric OCD (e.g., Leyton Obsessional Inventory—Child Version (LOI‐CV; Berg et al., 1986), Child Behavior Checklist—Obsessive–Compulsive Scale (CBCL‐OCS; Nelson et al., 2001), Obsessive–Compulsive Inventory—Child Version (OCI‐CV; Foa et al., 2010), Children's Obsessional Compulsive Inventory‐Revised‐Parent (ChOCI; Uher et al., 2008), Children's Florida Obsessive–Compulsive Inventory (C‐FOCI; Storch et al., 2009) are typically scored based on the presence or absence of symptoms. Specifically, the LOI‐CV and C‐FOCI have binary “yes”/“no” responses, while the CBC‐OCS, OCI‐CV, and ChOCI are scored on a three‐point Likert scale (perceived impairment is scored separately on the LOI‐CV, C‐FOCI, and ChOCI). While useful clinically, these scales either have limited response options that limits variance and/or creates severely skewed distributions, particularly in general population‐based or cross disorder samples. For example, the OCI‐CV subscales have skewed distributions in community samples, with only the obsessing subscale adequately discriminating between clinical and nonclinical youth with 62% classficiation accuracy (Rodríguez‐Jiménez et al., 2016). Similarly, the CBC‐OCS has a skewed distribution in nonclinical youth and psychiatric controls (Nelson et al., 2001). The TOCS overcomes the limitation of a truncated distribution as it is specifically designed to capture a wide distribution of OC trait scores using a strengths and weaknesses design on a seven‐point scale. The TOCS asks raters to score from strengths (−3; far less often than average) to weaknesses (+3; far more often than average). The TOCS has a six‐factor structure with strong psychometric properties in a community sample of children and adolescents, with scores on each factor being approximately normally distributed (Burton et al., 2018; Park et al., 2016). Thus, the TOCS captures more variance in OC traits than existing measures, with scores approximating a normal distribution, particularly in population samples.

In turn, the design of the TOCS makes it uniquely suited for genetic research and ensures strong psychometric properties. Quantitative OC trait research using the TOCS indicates that TOCS dimensions and total score are strongly heritable (30%–77%; Burton et al., 2018). Recently in a large community sample, the TOCS was used to identify the first genome‐wide significant variant for OC traits that was also associated with diagnosed OCD and to show shared polygenic risk between OC traits and OCD (Burton et al., 2021). Furthermore, in this same study we demonstrated that genome‐wide significance was lost when using OCD measures with the usual skewed distribution (e.g., CBCL‐OCS) (Burton et al., 2021). We have also shown that in a community sample, a cut‐off score of 0 on the TOCS showed adequate discrimination of self‐ and parent‐reported OCD cases from non‐cases (Park et al., 2016). These findings highlight the power of using rating scales that capture the full distribution of OC traits in community samples. What is currently unknown about the TOCS is how it performs in clinical samples. First, the diagnostic accuracy of the TOCS has yet to be evaluated using clinical samples with current OCD symptoms. Indeed, OCD symptoms have a variable course across the lifespan (Stewart et al., 2004), with a lifetime diagnosis not necessarily reflecting the presence of *current* symptomatology (i.e., patients may be asymptomatic). Second, the diagnostic accuracy of the TOCS for identifying OCD rather than co‐occurring disorders (e.g., autism spectrum disorder [ASD] and attention‐deficit/hyperactivity disorder [ADHD]; Burton et al., 2016; Kushki et al., 2019; van der Plas et al., 2016) needs to be established. For example, community‐reported ASD cases reported higher scores on the hoarding and symmetry/order dimensions of the TOCS than those with community‐reported OCD (Park et al., 2016). Ideally in a clinical sample, the TOCS will discriminate OCD cases from typically developing controls and will be able to discriminate these traits from symptoms of ADHD and ASD. A measure that has both strong diagnostic accuracy and the potential to inform quantitative research (including genetics) is beneficial for both clinicians and researchers by minimizing redundancy and burden for patients and their families.

The goal of this study is to confirm the psychometric properties of the TOCS in a clinical OCD sample and examine its sensitivity and specificity in discriminating OCD cases from controls, as well as from other neurodevelopmental disorders (ASD and ADHD). Specifically, we evaluated the factor structure, internal‐consistency reliability, convergent and divergent validity, and age and gender associations of the TOCS in a clinical sample of pediatric OCD. We examined the clinical ability of the TOCS to discriminate OCD cases from controls by splitting the OCD group into two subgroups (discovery and validation), in which a clinical cutoff was extracted in the discovery sample and confirmed in the validation sample. Lastly, we examined the clinical ability of the TOCS to discriminate OCD cases from clinical ASD and ADHD cases.

## METHOD

### Participants and procedure

Participants in the discovery sample of OCD and healthy controls were recruited from two tertiary care children's mental health clinics (SickKids, Toronto, Canada, and University of Michigan Medical Center, Ann Arbor, Michigan, USA). Participants in the validation sample with OCD, ADHD, and ASD diagnoses or typically developing controls were recruited from tertiary care clinics as part of the Province of Ontario Neurodevelopmental Disorders (POND) Network (Ontario, Canada). TOCS total scores did not significantly vary based on recruitment site for the OCD group: *F*(3, 346) = 2.46, *p* = .062. Informed consent (and verbal assent when applicable) was obtained before research participation. Ethics approval was obtained from the relevant institutions listed above.

Participants ranged in age from 6 to 21 years old and had a primary diagnosis of OCD (*n* = 350), ADHD (*n* = 820), or ASD (*n* = 794) or were typically developing control participants (*n* = 391). Diagnoses for the clinical groups were based on diagnostic criteria from the DSM‐IV or DSM‐5 following a rigorous, semi‐structured clinical assessment with a psychologist or psychiatrist. The following gold‐standard assessment tools were used to confirm the primary diagnoses for each clinical group: the Kiddie Schedule for Affective Disorders and Schizophrenia (K‐SADS (Kaufman et al., 1997)), and/or the Children's Yale‐Brown Obsessive–Compulsive Scale (CY‐BOCS; Scahill et al., 1997), and/or the Scheduled for Obsessive–Compulsive and Other Behavioral Syndromes (SOCOBS; Rough et al., 2020) for OCD; the Parent Interview for Child Symptoms (PICS; Ickowicz et al., 2006) for ADHD; and the Autism Diagnostic Observation Schedule‐2 (ADOS‐2; Lord et al., 2000), and/or the Autism Diagnostic Interview‐revised (ADI‐R; Rutter et al., 2003) for ASD. Clinical group membership was determined based on the primary diagnosis assigned during the assessment (i.e., participants were not excluded based on comorbid diagnoses^1^). Additionally, participants were included in the OCD group only if they currently met criteria for OCD at the time of assessment (i.e., excluded participants with lifetime symptoms only). Participant demographics are presented in Table 1.

**TABLE 1:** Participant demographics by diagnosis

	*N*	Average age (years)	Gender (% female)
OCD	350	13.04 ± 3.02	50%
Discovery	166	12.86 ± 3.17	45%
Validation	184	13.11 ± 2.89	50%
Controls	391	13.26 ± 3.93	51%
Discovery	164	14.12 ± 3.52	59%
Validation	227	12.86 ± 3.94	45%
ADHD	820	10.44 ± 2.85	26%
ASD	794	11.71 ± 3.58	22%

*Note*: The OCD discovery and validation samples did not differ in terms of age *t*(348) = 0.77, SE = 0.32, *p* = .44 or gender, *x* ^2^(1, *N* = 350) = 0.81, *p* = .37. For the control sample, the discovery sample was significantly older than the validation sample, *t*(389) = 3.26, SE = 0.39, *p* < .01, and consisted of more females, *x* ^2^(1, *N* = 391) = 7.69, *p* < .05. Consideration of age and gender did not affect clinical cut‐offs (see ROC analyses and Appendix S2 for details).

Abbreviations: ADHD, attention‐deficit/hyperactivity disorder; ASD, autism spectrum disorder; OCD, obsessive–compuslsive disorder.

### Measures

#### Toronto Obsessive–Compulsive Scale

The TOCS is a 21‐item measure of OC traits over the past 6 months that is specifically designed to capture a wide distribution of responses (Park et al., 2016). Parents report the extent to which their child engages in each of the 21 OC thoughts or behaviors using a seven‐point Likert scale (−3 = far less often than average, 0 = average, +3 = far more often than average). The TOCS assesses a range of common OC symptoms. It includes the following 6 subscales derived from a factor analysis conducted by our group in a large pediatric community sample: Counting/checking, cleaning/contamination, hoarding, symmetry/order, rumination, and superstition (Burton et al., 2018; Park et al., 2016). The scale has strong reliability and validity in community‐based samples of children and adolescents (Burton et al., 2018; Park et al., 2016). The TOCS is freely accessible online: https://lab.research.sickkids.ca/schachar/resources‐and‐tools/#tools.

#### Child Behavior Checklist—Obsessive–Compulsive Scale

The CBCL‐OCS (Nelson et al., 2001) was used to examine convergent validity of the TOCS. The CBCL‐OCS is an eight‐item parent‐report screening tool used to assess symptoms of OCD. Parents report the extent to which their child experiences each symptom on a 0–2 scale, with higher scores indicating greater symptoms. The CBCL‐OCS is reliable and valid as a screening tool for pediatric OCD (Hudziak et al., 2006; Nelson et al., 2001).

#### Strengths and weaknesses of ADHD Symptoms and Normal Behavior Rating Scale

The strengths and weaknesses of ADHD Symptoms and Normal Behavior Rating Scale (SWAN) (Swanson et al., 2012) measures ADHD symptoms and was used to assess the divergent validity of the TOCS. The SWAN is a 18‐item scale in which parents rate the extent to which their child engages in each behavior on a −3 to +3 Likert scale. The SWAN is reliable and valid in pediatric samples, with high SWAN scores associated with polygenic risk for ADHD (Burton et al., 2019). Scores are reversed with higher scores reflecting increased traits of ADHD.

#### Social Communication Questionnaire

The Social Communication Questionnaire (SCQ) (Rutter et al., 2003) measures social communication and behaviors associated with ASD and was also used to assess the divergent validity of the TOCS. The SCQ is a 40‐item scale in which parents rate the extent to which their child engages in each behavior, with higher scores indicating greater symptom severity. The *current* symptoms (not lifetime) version of the SCQ was used in the current study.

### Data analysis

All data analyses were conducted using R v4.0.2. Confirmatory factor analysis (CFA) (Rosseel, 2012) was used to examine the factor structure of the TOCS in the OCD group. Two indices of reliability (Jorgensen et al., 2020) were examined: Cronbach's alpha and Omega coefficient. Measurement invariance (Rosseel, 2012) was tested to determine if the same factor structure held across groups. Measurement invariance was accepted if the change in model fit was <0.01 (Putnick & Bornstein, 2016).

Given the unique distribution of the TOCS (i.e., it has negative scores and a much wider range of scores than many other screening tools), we examined the performance of the TOCS total score (summed total), TOCS symptom count (number of items with scores ≥2), and the TOCS max average (highest raw average score within a subscale). Average scores were computed for all six TOCS subscales. Pearson correlations were used to examine convergent and divergent validity using these TOCS indices within the OCD sample.

ROC analyses (Khan & Brandenburger, 2020) were used to identify the optimal cut‐points for discriminating those with a clinical diagnosis of OCD from controls. The area under the ROC curve (AUC) indicates the overall accuracy of discrimination, with higher values indicating better discrimination of cases from controls (AUC ≥0.90 indicates excellent discrimination, ≥0.80 good discrimination (Zhu et al., 2010)). The Youden index was used to determine the optimal cut‐point from the ROC curve. ROC analyses were conducted for the TOCS total score, symptom count, and maximum average score. Established cut‐offs from the discovery sample were applied in the independent validation sample. Across both samples, we report the sensitivity, specificity, and accuracy (overall probably of correct classification, based on the proportion of true positives [TP] and true negatives [TN]) of the TOCS at these cutoffs. We also examined whether the ROC results differed by age and gender, as well as whether they differed when the Hoarding factor was not included.

Additionally, we examined how well the TOCS was able to discriminate between clinical groups (OCD, ADHD, ASD) using ROC. We report the AUC, which reflects the overall ability of the TOCS to discriminate OCD from ADHD, as well as OCD from ASD. We also report the classification accuracy (overall probability of correct classification as OCD and non‐OCD) at the previously identified cutoff scores. Lastly, we tested whether TOCS scores significantly varied by clinical group. Tukey HSD tests were used for post‐hoc comparisons and effects sizes are reported using Cohen's *d*.

## RESULTS

### Confirmatory factor analysis in OCD sample

The previously identified six‐factor model (Park et al., 2016) was tested using the full OCD clinical sample (*N* = 350). The model demonstrated adequate fit to the data, *χ* ^2^ (137) = 596.60, CFI = 0.897, RMSEA = 0.098 90% CI [0.090, 0.106], SRMR = 0.066. Factor loadings are presented in Table S1. The model fit significantly improves when the strictness of the model is relaxed using an exploratory structural equation modeling (ESEM) approach, which allows cross‐loadings (see Appendix S1 and Table S2).

Latent factor correlations are presented in Table S3. Overall, all factors were positively correlated with one another. The strongest correlations were observed between the counting/checking, symmetry/order, superstition, and rumination factors. Cleaning/contamination and hoarding tended to have weaker correlations with the other factors.

### Reliability in OCD sample

All TOCS subscales demonstrated acceptable to strong levels of internal consistency (Table S1). The TOCS total score demonstrated strong internal consistency, *α* = 0.91, *ω* = 0.95.

### Convergent and divergent validity in OCD sample

#### Convergent validity

TOCS total scores and subscales demonstrated small to moderate positive correlations with the CBCL‐OCS, with the exception of hoarding which was not significantly related to the CBCL‐OCS (Table 2).

**TABLE 2:** Bivariate correlations between the TOCS and existing measures

	TOCS total	TOCS symptom count	TOCS max average	Counting/checking	Cleaning/contamination	Hoarding	Symmetry/order	Superstition	Rumination
CBCL‐OCS	0.34***	0.42***	0.40***	0.27***	0.14*	0.10	0.21***	0.30***	0.46***
SWAN	0.10	0.10	0.10	0.07	0.02	0.24***	0.18**	0.08	0.05
SCQ	0.15**	0.23**	0.17**	0.10	0.16**	0.14*	0.16**	0.03	0.07
Age	−0.13*	−0.14*	−0.16**	−0.15**	0.04	−0.18**	−0.11*	−0.08	−0.06
Gender	−0.01	−0.02	−0.10	−0.05	−0.06	−0.02	0.04	−0.01	0.03

*Note*: Gender was scored male = 1, female = 2.

Abbreviations: CBCL‐OCS, Child Behavior Checklist—Obsessive–Compulsive Scale; SCQ, Social Communication Questionnaire; SWAN, Symptoms and Normal Behavior Rating Scale; TOCS, Toronto Obsessive–Compulsive Scale.

***p < .001, **p < .01, *p < .05.

#### Divergent validity

TOCS total scores were not significantly related to the SWAN; however, small positive correlations were found with the Hoarding and Symmetry/order factors (Table 2). TOCS total scores and the cleaning/contamination, hoarding, and symmetry/order factors demonstrated small positive correlations with the SCQ.^2^

### Age and gender differences in OCD sample

There were small negative correlations between age and TOCS scores but no significant gender differences in TOCS scores (Table 2). We also explored whether age and gender interacted to predict TOCS scores. A significant age by gender interaction was found only for the TOCS max average score, *β* = −0.08, SE = 0.04, *p* = .03. For girls only, there was a negative association between age and TOCS max average scores, *β* = −0.09, SE = 0.03, *p* = .001. The association between age and TOCS max average scores among boys was non‐significant, *p* = .61. A similar pattern was observed using the CBCL‐OCS: for girls only, there was a negative association between age and CBCL‐OCS scores, *β* = −0.27, SE = 0.09, *p* = .003.

### ROC analyses in OCD sample

For the ROC analyses, the OCD and control samples were divided into a *discovery sample* (i.e., used to determine a cut‐point) and a *validation sample* (i.e., used to test the cut‐point). The discovery sample consisted of data from the two tertiary care children's mental health clinics (*n* = 166 OCD, *n* = 164 controls), whereas the validation sample consisted of data from POND (*n* = 184 OCD, *n* = 227 controls). Comorbidities were as follows within the OCD discovery sample: 12.7% tic disorder, 33% anxiety disorders, 4.2% ASD, 21.7% ADHD, 11.4% mood disorders.

In the discovery sample, the AUC [95% CI] for all TOCS indices excellent diagnostic discrimination of cases from controls: TOCS total score = 0.95, [0.93, 0.97], TOCS symptom count = 0.96, [0.94, 0.98], TOCS max average = 0.96, [0.94, 0.98]. A TOCS total score of 1, a symptom count of 2, and a max average score of 1 maximized sensitivity and specificity (Table 3). Age and gender did not affect the cutoffs, nor did the exclusion of the hoarding dimension (see Appendix S1 and Table S4). The TOCS maintained an excellent level of diagnostic discrimination in the validation sample, with overall classification accuracy ≥85% across scoring approaches (Table 3).

**TABLE 3:** Sensitivity and specificity analyses

	Cutoff score	Discovery sample			Validation sample		
		Accuracy	Sensitivity	Specificity	Accuracy	Sensitivity	Specificity
TOCS total score	1	87%	0.77	0.98	85%	0.78	0.91
TOCS symptom count	2	93%	0.90	0.96	90%	0.87	0.93
TOCS max average	1	92%	0.92	0.92	88%	0.88	0.88

Abbreviation: TOCS, Toronto Obsessive–Compulsive Scale.

### Discriminating clinical groups

The TOCS demonstrated good diagnostic discrimination of OCD cases from ADHD cases, AUC [95% CI]: TOCS total score = 0.86, [0.84, 0.89], TOCS symptom count = 0.87, [0.84, 0.89], TOCS max average = 0.81, [0.78, 0.84]. Using the cutoff scores obtained above (TOCS total = 1, symptom count = 2, and max average = 1), classification accuracy for OCD and ADHD were as follows: TOCS total score = 77%, TOCS symptom count = 73%, and TOCS max average = 66%.

When discriminating OCD cases from ASD cases, the AUC [95% CI] was acceptable for the TOCS total score = 0.81, [0.78, 0.84] and the TOCS symptom count = 0.80, [0.77, 0.83]. Diagnostic discrimination between OCD and ASD was poor when using the TOCS max average = 0.75, [0.71, 0.78]. Using the cutoff scores obtained above (TOCS total = 1, symptom count = 2, and max average = 1), classification accuracy for OCD and ASD were as follows: TOCS total score = 71%, TOCS symptom count = 62%, and TOCS max average = 56%.

### Group comparisons across TOCS scores

Measurement invariance of the TOCS was examined before making group comparisons. The clinical groups and control sample were combined for invariance testing for a total sample size of *N* = 2362. The TOCS demonstrated measurement invariance at the configural, metric, and scalar levels, indicating equivalent item loadings, and intercepts across groups (Table S5).

We examined whether TOCS total scores differed by clinical group using ANOVAs (Bonferroni corrected *p* = .016). There was a significant effect of group on TOCS total raw score, *F*(2, 1961) = 264.40, *p* < .001, *η* ^2^ = 0.21; symptom count, *F*(2, 1961) = 367.80, *p* < .001, *η* ^2^ = 0.27; and subscale maximum average *F*(2, 1961) = 151.20, *p* < .001, *η* ^2^ = 0.13. Post hoc comparisons using Tukey HSD tests indicated that across all indices, the OCD group had significantly higher scores than the ADHD group (all *p* < .001, Cohen's *d* ≥ 1.13) and ASD group (all *p* < .001, Cohen's *d* ≥ 0.86). Additionally, the ASD group had significantly higher TOCS scores across indices than the ADHD group, all *p* < .001, Cohen's *d* ≥ 0.29. See Table 4 for TOCS total scores and standard deviations by clinical group, including scores for the typically developing controls.

**TABLE 4:** TOCS total scores and subscale averages by group

	TOCS total score	TOCS symptom count	TOCS max average	Counting/checking	Cleaning/contamination	Hoarding	Symmetry/order	Superstition	Rumination
OCD	12.40 (22.20)	7.27 (4.64)	2.06 (1.05)	0.64 (1.51)	0.75 (1.56)	0.12 (1.73)	0.73 (1.45)	0.13 (1.46)	0.99 (1.61)
ADHD	−24.40 (26.10)	1.63 (2.61)	0.34 (1.69)	−1.45 (1.45)	−1.27 (1.49)	−0.33 (1.82)	−0.97 (1.57)	−1.67 (1.34)	−0.79 (1.72)
ASD	−15.40 (25.30)	2.65 (3.23)	0.82 (1.60)	−0.76 (1.52)	−0.92 (1.45)	−0.23 (1.91)	−0.12 (1.61)	−1.52 (1.41)	−0.55 (1.81)
Controls	−39.0 (24.0)	0.26 (1.14)	−1.05 (1.51)	−2.16 (1.21)	−1.77 (1.30)	−1.59 (1.48)	−1.81 (1.29)	−2.17 (1.18)	−1.67 (1.43)

*Note*: Standard deviations are presented in brackets. All group means are significantly different at *p* < .001 with the exception of Hoarding scores, which did not significantly differ between OCD, ADHD, and ASD groups.

Abbreviations: ADHD, attention‐deficit/hyperactivity disorder; ASD, autism spectrum disorder; TOCS, Toronto Obsessive–Compulsive Scale.

A one‐way MANOVA was used to examine the effect of group (OCD, ADHD, and ASD) on TOCS subscale scores. MANOVA results are reported using Pillai's trace as the homogeneity of covariances assumption was violated, Box's *M* = 287.1, *p* < .001. Levene's test of homogeneity of variances was violated for cleaning/contamination scores only, *p* = .12; thus, results are reported using Welch's ANOVA.

**FIGURE 1:**
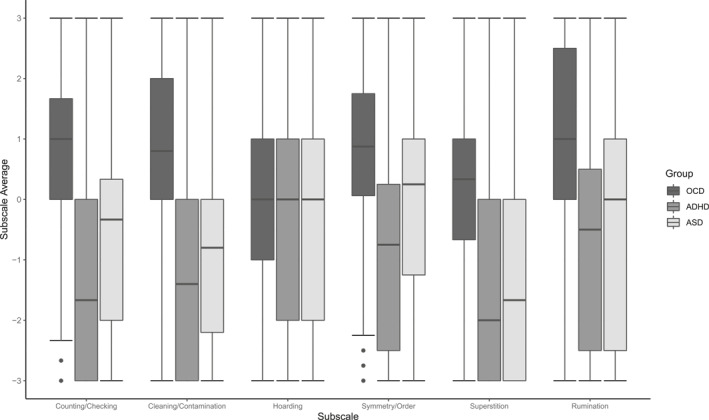
Boxplots depicting Toronto Obsessive–Compulsive Scale subscale scores by clinical group

TOCS subscale scores significantly differed based on group membership, *F*(12, 3864) = 71.91, Pillai's trace = 0.37, *p* < .001. One‐way ANOVA models were used to examine the effect of group membership on each subscale score separately (Bonferroni corrected *p* = .0083). With the exception of hoarding, there was a significant effect of group membership on all subscale average scores, all *p* < .001. Post‐hoc comparisons indicated that across all TOCS subscales (except hoarding) the OCD group reported the highest subscale scores, followed by the ASD group, and then by the ADHD group, respectively (Table 4). As shown in Figure 1, counting/checking and cleaning/contamination tended to be the most distinct to the OCD group, whereas the symmetry/order and rumination subscales demonstrated some overlap with the ASD group. Hoarding levels were not significantly different across the three clinical groups, *F*(2, 1953) = 7.23, *p* = .0088.

## DISCUSSION

There is a need to develop quantitative trait‐based measures to facilitate research exploring the etiology of mental health disorders, including pediatric OCD and particularly in community‐based studies. At the same time, such measures must also be clinically valid and will have the most meaningful impact if they are also helpful for clinicians, patients, and their families. Results from the current research indicate that the parent‐report version of the TOCS is a reliable and valid rating scale within clinical samples of pediatric OCD. As hypothesized, the six‐factor structure (Burton et al., 2018; Park et al., 2016) of the TOCS was confirmed, with each factor demonstrating strong internal consistency, convergent validity with the CBCL‐OCS, and divergent validity with the SWAN and SCQ.

All TOCS composite scores (i.e., TOCS total score, symptom count, maximum subscale average) demonstrated excellent ability to discriminate OCD cases from controls, highlighting the robustness of this measure. Indeed, the sensitivity and specificity of the TOCS is just as strong—if not somewhat stronger—than existing screening tools for OCD such as CBCL‐OCS (Nelson et al., 2001). Moreover, the TOCS captures a wider variety of OC traits compared with the CBCL‐OCS, which was derived from a broader symptom measure and contains items that were not designed to assess OC symptoms specifically. Interestingly, the TOCS symptom count measure performed as well as the TOCS total score in terms of discriminating OCD cases. It is important to note that this does not negate the strengths/weaknesses design of the TOCS, as a wide distribution of scores remains important for genetic and cross‐disorder research (Burton et al., 2021). Rather, it highlights that the same measure can easily be used by both researchers and clinicians. Clinicians may wish the TOCS symptom count score cutoff (two items ≥2) to screen for pediatric OCD, as this indicator is quickly scored and is a highly sensitive and specific indicator of clinical levels of OC traits. Moreover, the TOCS symptom count did not significantly differ by age or gender, thereby facilitating interpretation as the same cut‐off score of two symptoms can be used for all patients.

While TOCS total scores and symptom counts did not differ by age or gender within the clinical OCD sample, there was a significant age by gender interaction for TOCS maximum subscale average score. Older girls had lower TOCS maximum average scores than younger girls, whereas no age difference was observed for boys. This does not appear to be an issue specific to the TOCS as the same age by gender interaction was found using the CBCL‐OCS. This finding was unexpected as the developmental prevalence of pediatric OCD often follows a different gender pattern—the prevalence of OCD among males is higher before puberty, whereas OCD becomes more prevalent among females after puberty (Mathes et al., 2019). There are several possible reasons for this trend. First, the average age of the OCD sample was 13 years old, suggesting that a large proportion of the sample was pre‐pubescent or just entering pubertal developmental. Additionally, the age and gender patterns observed in the current study may be an artifact of relying on parent‐report. It is possible that older girls are better at disguising their symptoms from parents or engage in more mental rituals (as opposed to overt, behavioral compulsions) than younger girls. Future research should compare the self‐ and parent‐report versions of the TOCS to better understand this gender difference. Nonetheless, the strong sensitivity and specificity of the parent‐report version of the TOCS indicates that parents are reliable and valid informants of pediatric OC traits.

As expected, parents of patients with a primary diagnosis of OCD reported significantly higher TOCS scores across all indices (except hoarding) than parents of patients with primary diagnoses of ADHD or ASD. These findings suggest that the TOCS specifically measures OC traits, not simply general psychopathology. While OCD cases had the highest TOCS scores on average across dimensions, it is important to note that many parents of children and adolescents with ADHD and ASD also reported elevated OC traits. In particular, there was a high degree of overlap between the OCD and ASD groups on the symmetry/order and rumination dimensions, which would be expected given the phenotypic similarity (Kushki et al., 2019) and elevated comorbidity rates (van der Plas et al., 2016) between these disorders. In contrast, other dimensions, such as counting/checking and cleaning/contamination, were more specific to the OCD cases. A small previous study comparing children with ASD to children with OCD similarly reported that checking and contamination were relatively specific to the latter group (Ruta et al., 2009). Previous research using a community sample found that community‐reported ASD cases had significantly higher symmetry/ordering and hoarding TOCS scores than community‐reported OCD cases (Park et al., 2016). It is possible that many of the community‐reported OCD cases in the Park et al. (2016) study were not experiencing current symptomology (given that OCD symptoms wax and wane across the life course, whereas children with ASD (which does not typically wax and wane) had persistent symmetry/ordering and hoarding symptoms, resulting in higher subscale scores for this group.

The hoarding dimension of the TOCS demonstrated a unique pattern of results. Consistent with contemporary conceptualizations of hoarding (Burton et al., 2016; Grisham et al., 2005; Rachman et al., 2009) as a separate diagnosis from OCD within obsessive‐compulsive and related disorders in DSM‐5, findings from the current research suggest that hoarding is distinct from OCD. Within the OCD sample, the hoarding dimension was not significantly correlated with the CBCL‐OCS, and the same clinical cutoffs were obtained with and without hoarding scores. Additionally, unlike all other TOCS dimensions, hoarding scores did not significantly differ between the OCD, ADHD, and ASD groups. Elevations on the hoarding factor alone may indicate that further assessment of OCD, ADHD, and ASD are warranted. Together, these results add further support differentiating hoarding from OCD, and support previous research highlighting the comorbidity between hoarding, ADHD, and ASD (Burton et al., 2016; Morris et al., 2016). Future research should consider the clinical utility of the TOCS hoarding dimension for discriminating clinical cases of hoarding disorder.

The current research is the first to evaluate the TOCS using a large pediatric clinic sample and should be considered with the following limitations. The TOCS has both parent‐ and self‐report formats; however, we only evaluated the parent‐report version. Additional research is necessary to establish the psychometric properties and clinical cut‐offs of the self‐report TOCS, which may be especially useful for older adolescent samples. While we report several indices of diagnostic accuracy (e.g., sensitivity, specificity, AUC), the Youden index was used to select cut‐points for the TOCS, which is not sensitive to differences in sensitivity and specificity (Šimundić, 2009). Additionally, our research examined discriminant validity of the TOCS between OCD, ADHD, and ASD clinic samples. It is unknown the degree to which the TOCS and its dimensions can discriminate between OCD and other comorbid psychiatric disorders, such as anxiety disorders or eating disorders. Indeed, the clinical groups in the current study were based only on *primary diagnosis*, which is the diagnosis that the assessing clinician felt best captured the patient's presenting concerns. In other words, we did not exclude participants from the ADHD or ASD groups who may have had comorbid OCD. While we believe this adds to the ecological validity of our findings, it is possible that the discriminant validity of the TOCS would in fact be stronger with “pure” clinical groups. Additionally, we did not include very young children in our sample (i.e., <6 years old). Given that many ASD diagnoses are assigned during the preschool years, it is possible that discriminant classification of ASD from OCD cases may be different in such age groups. Lastly, the current research is cross sectional and specifically examined OCD cases with current symptomology (i.e., cases with a past history of OCD but no current symptoms were excluded). Future research may wish to examine how TOCS scores vary over time.

In conclusion, our results build upon previous research with community samples (Burton et al., 2018; Park et al., 2016) by demonstrating the strong psychometric properties of the TOCS in a clinical OCD sample. The parent‐report TOCS is a reliable and valid measure that has excellent diagnostic accuracy for identifying clinical levels of OC traits in pediatric clinic samples. By establishing the TOCS both as a meaningful tool for genetic research and as a clinically valid rating scale, researchers and clinicians may be able to simultaneously gather data to understand the etiology of OCD, as well as data to inform clinical assessments.

## CONFLICT OF INTERESTS

The authors have declared that they have no competing or potential conflicts of interest.

## ETHICS STATEMENT

Ethics approval was obtained from the relevant institutions.

## Supporting information

Supporting Information S1

## Data Availability

The data that support the findings of this study are available from the corresponding author upon reasonable request.

## ENDNOTES

^1^
Comorbidity rates for the ADHD group were as follows: 4.3% ASD, 3.8% OCD, 16.0% anxiety, 2.8% intellectual disability. Comorbidity data for the ASD group was only available for a subset of the sample (*n* = 300) and were as follows: 21.7% ADHD, 2.3% OCD, 11.7% anxiety, and 21.1% intellectual disability.

^2^
For 47 cases only lifetime SCQ scores were available. The pattern of results does not change whether or not these cases are included in the correlation matrix.
