# Supplementary material for: Clinical validation of the parent‐report Toronto Obsessive–Compulsive Scale (TOCS): A pediatric open‐source rating scale
Source: JCPP Adv. 2021 Dec 3;1(4):e12056. doi: 10.1002/jcv2.12056 (PMC10242913; doi:10.1002/jcv2.12056)
Supplement: Supplementary file 1 — Supporting Information S1 [file JCV2-1-e12056-s001.docx]

**Appendix S1. Supplementary Data**

Table S1. Factor loadings and reliabilities

| TOCS Latent Factor and Items | Factor Loading |
| --- | --- |
| *Factor 1. Counting/Checking* (α = 0.78, ω = .78) |  |
| 2. “Do certain” | .78 |
| 3. “Checks” | .67 |
| 6. “Count” | .77 |
| *Factor 2. Cleaning /Contamination* (α = 0.92, ω = .93) |  |
| 13. “Dirt” | .84 |
| 14. “Germs” | .89 |
| 15. “Wash” | .91 |
| 16. “Clean” | .87 |
| 17. “Ruined” | .72 |
| *Factor 3. Hoarding* (α = 0.92, ω = .92) |  |
| 18. “Useless” | .97 |
| 19. “Throwing” | .88 |
| *Factor 4. Symmetry/Order* (α = 0.86, ω = .85) |  |
| 7. “Symmetry” | .70 |
| 10. “Interfere” | .74 |
| 11. “Repeat” | .85 |
| 12. “Not exactly” | .80 |
| *Factor 5. Superstition* (α = 0.71, ω = .73) |  |
| 9. “Special” | .75 |
| 20. “Bad luck” | .80 |
| 21. “Healthy” | .50 |
| *Factor 6. Rumination* (α = 0.90, ω = .90) |  |
| 4. “Guilty” | .87 |
| 5. “Thinking” | .94 |

*Note*. Standardized factor loadings are presented. Consistent with Park et al., 2016, items 1 and 8 were not included in the factor model. All factor loadings were statistically significant at *p* < .001.

Table S2. TOCS factor loadings for ESEM results

|  | F1 – Counting/ Checking | F1 – Cleaning/ contamination | F3 – Hoarding | F4 – Symmetry/ Order | F5 - Superstition | F6 - Rumination |
| --- | --- | --- | --- | --- | --- | --- |
| TOCS2 | **.71***** |  |  |  |  |  |
| TOCS3 | **.45***** | .06 | .13* | .06 | -.02 | .24*** |
| TOCS4 | .04 | -.03 | .02 | -.06 | .05 | **.82***** |
| TOCS5 |  |  |  |  |  | **.97***** |
| TOCS6 | **.41***** | -.07 | .05 | .27*** | .25** | -.01 |
| TOCS7 | .13 | -.07 | -.08 | **.65***** | .09 | .06 |
| TOCS9 | -.05 | .09 | -.11 | .16* | **.76***** | -.03 |
| TOCS10 |  |  |  | **.81***** |  |  |
| TOCS11 | **.80***** | .07 | -.06 | **.47***** | -.28* | -.024 |
| TOCS12 | **.37***** | .12* | .003 | **.53***** | -.27** | **.36***** |
| TOCS13 | .09 | **.79***** | -.03 | .16 | -.11 | .03 |
| TOCS14 |  | **.92***** |  |  |  |  |
| TOCS15 | .06 | **.91***** | -.07 | -.04 | .05 | -.04 |
| TOCS16 | .16* | **.86***** | -.08 | .06 | -.11 | .04 |
| TOCS17 | .14 | **.65***** | .12* | .17* | .01 | -.13* |
| TOCS18 | .06 | -.02 | **.85***** | .01 | .05 | -.01 |
| TOCS19 |  |  | **.96***** |  |  |  |
| TOCS20 |  |  |  |  | **.72***** |  |
| TOCS21 | -.18 | .27*** | .04 | -.08 | **.39***** | .27*** |

*Note.* Standardized factor loadings. Salient factor loadings (≥ .30) are bolded.

*** *p* < .001, ** *p* < .01, * *p* < .05

The ESEM model demonstrated good fit to the data, χ^2^ (72) = 213.19, CFI = .967, RMSEA = .077 (90% CI .065, .089), SRMR = 0.021. Notable cross-loadings were found for item 11 (“has to repeat actions before they seem quite right”) and item 12 (“worries a lot if he/she does something not exactly the way he/she likes”). These cross-loadings are consistent with theoretical overlap between TOCS dimensions.

Table S3. Latent factor correlations

|  | 1. | 2. | 3. | 4. | 5. | 6. |
| --- | --- | --- | --- | --- | --- | --- |
| 1. Counting / checking | - |  |  |  |  |  |
| 2. Cleaning / contamination | .29 | - |  |  |  |  |
| 3. Hoarding | .52 | .17 | - |  |  |  |
| 4. Symmetry / order | .88 | .38 | .51 | - |  |  |
| 5. Superstition | .85 | .31 | .47 | .67 | - |  |
| 6. Rumination | .64 | .14 | .32 | .47 | .61 | - |

*Note.* All correlations were statistically significant at *p* < .001.

**Appendix S2. Supplemental ROC analyses**

Given that older girls tended to have lower scores on the TOCS max average index, we also explored whether this cut-off score differed by age and gender. Among younger girls (**≤**12 years old: OCD n = 68, controls n = 80), a max average score of 1.2 was the best determinant of OCD case-control status. Among older girls (>12 years old: OCD n = 99, controls n = 121), a max average score of 1 was the best determinant of OCD case-control status.

ROC analyses were also examined without the hoarding dimension in the TOCS total score, symptom count, and max average. In the discovery sample, AUC for discriminating OCD cases from controls was excellent for all TOCS indices: TOCS total score = .95, [.93, .98], TOCS symptom count = .96, [.94, .98], TOCS max average = .97, [.96, .99]. The same cut-off values maximized both sensitivity and specificity, with excellent overall accuracy rates (Table S4). Using these cut-off values, a high level of diagnostic discrimination of OCD cases from controls was replicated in the validation sample.

Table S4. Sensitivity and specificity analyses without hoarding

|  |  | *Discovery sample* | | | *Validation sample* | | |
| --- | --- | --- | --- | --- | --- | --- | --- |
|  | Cut-off score | Accuracy | Sensitivity | Specificity | Accuracy | Sensitivity | Specificity |
| TOCS total score (no hoarding) | 1 | 87% | .77 | .98 | 86% | .79 | .92 |
| TOCS symptom count (no hoarding) | 2 | 94% | .90 | .98 | 91% | .87 | .93 |
| TOCS max average (no hoarding) | 1 | 94% | .89 | .96 | 89% | .85 | .93 |

Additionally, we examined how well the TOCS (without hoarding) was able to discriminate between clinical groups. The TOCS (without hoarding) demonstrated good diagnostic discrimination of OCD cases from ADHD cases, AUC [95%CI]: TOCS total score = .87, [.85, .90], TOCS symptom count = .88, [.86, .91], TOCS max average = .87, [.84, .89]. Using the cut-off scores obtained above, (TOCS total = 1, symptom count = 2, max average =1), the overall accuracy rates for OCD and ADHD were as follows: TOCS total score = 79%, TOCS symptom count = 78%, TOCS max average = 75%.

When discriminating OCD cases from ASD cases, the AUC [95%CI] was acceptable for the TOCS total score = .82, [.79, .85] and the TOCS symptom count = .82, [.79, .85]. Diagnostic discrimination between OCD and ASD was borderline when using the TOCS max average = .79, [.76, .82]. Using the cut-off scores obtained above, (TOCS total = 1, symptom count = 2, max average =1), the overall accuracy rates for OCD and ASD were as follows: TOCS total score = 73%, TOCS symptom count = 66%, TOCS max average = 63%.

Table S5. TOCS measurement invariance across OCD, ADHD, ASD, and control groups.

|  | X^2^ (df) | RMSEA [90%CI] | CFI | SRMR |
| --- | --- | --- | --- | --- |
| *Invariance models* |  |  |  |  |
| Configural | 3222.10 (548) | .094 [.091, .097] | .922 | .057 |
| Metric | 3512.60 (587) | .091 [.088, .094] | .921 | .060 |
| Scalar | 4100.90 (626) | .096 [.094, .099] | .907 | .064 |
|  | X^2^ (df) | Δ RMSEA | Δ CFI | Δ SRMR |
| *Model comparisons* |  |  |  |  |
| Configural vs. metric | 90.5 (39) | .003 | .001 | .003 |
| Metric vs. scalar | 588.30 (39) | .005 | .014 | .004 |
